# Differential Effects of Topical Vitamin E and C E Ferulic® Treatments on Ultraviolet Light B-Induced Cutaneous Tumor Development in Skh-1 Mice

**DOI:** 10.1371/journal.pone.0063809

**Published:** 2013-05-14

**Authors:** Erin M. Burns, Kathleen L. Tober, Judith A. Riggenbach, Donna F. Kusewitt, Gregory S. Young, Tatiana M. Oberyszyn

**Affiliations:** 1 Department of Pathology, The Ohio State University, Columbus, Ohio, United States of America; 2 Department of Molecular Carcinogenesis, Science Park, The University of Texas MD Anderson Cancer Center, Smithville Texas, United States of America; 3 Center for Biostatistics, The Ohio State University, Columbus, Ohio, United States of America; Columbia University Medical Center, United States of America

## Abstract

Because of the ever-increasing incidence of ultraviolet light B (UVB)-induced skin cancer, considerable attention is being paid to prevention through the use of both sunscreens and after sun treatments, many of which contain antioxidants. Vitamin E is included as an antioxidant in many sunscreens and lotions currently on the market. Studies examining the efficacy of vitamin E as a topical preventative agent for UVB-induced skin cancer have yielded conflicting results. A likely contributor to differences in study outcome is the stability of vitamin E in the particular formulation being tested. In the current study we examined the effects of topical vitamin E alone as well as vitamin E combined with vitamin C and ferulic acid in a more stable topical formula (C E Ferulic®). Mice were exposed to UVB for 10 weeks in order to induce skin damage. Then, before the appearance of any cutaneous lesions, mice were treated for 15 weeks with a topical antioxidant, without any further UVB exposure. We found that topical C E Ferulic decreased tumor number and tumor burden and prevented the development of malignant skin tumors in female mice with chronically UVB-damaged skin. In contrast, female mice chronically exposed to UVB and treated topically with vitamin E alone showed a trend towards increased tumor growth rate and exhibited increased levels of overall DNA damage, cutaneous proliferation, and angiogenesis compared to vehicle-treated mice. Thus, we have demonstrated that topical 5% alpha tocopherol may actually promote carcinogenesis when applied on chronically UVB-damaged skin while treating with a more stable antioxidant compound may offer therapeutic benefits.

## Introduction

Over two million people are diagnosed with a form of non-melanoma skin cancer (NMSC) each year in the United States, making skin cancer more prevalent than all other cancers combined [1]. Squamous cell carcinoma (SCC), a malignant form of NMSC, makes up about 16% of all skin cancers. While the mortality rate from SCC is relatively low–about 3000 deaths per year–SCC can be quite disfiguring since most lesions are located on sun exposed body parts such as the face and arms. Additionally, topical therapies used to treat precursor actinic keratotic lesions are not effective on invasive SCC, necessitating invasive surgeries. With the increasing NMSC incidence, there has been a renewed focus on sunscreens and other methods of preventing skin cancer, including antioxidant supplementation in food, sunscreens, and lotions.

Following UVB exposure, both infiltrating inflammatory cells and activated epidermal keratinocytes generate reactive oxygen species (ROS). Endogenous antioxidants play an important role in detoxifying ROS and maintaining cutaneous homeostasis. If ROS levels overwhelm the cutaneous antioxidant networks, the cells will be subjected to oxidative stress [2]. Major mechanisms by which ROS foster skin tumor development include induction of DNA damage [3], [4], inflammation [5], and angiogenesis [6], [7], [8].

Endogenous antioxidants in the skin include the enzyme catalase as well as ascorbic acid and alpha tocopherol (vitamins C and E, respectively) [9], [10], [11], [12], [13]. Catalase, the main cutaneous antioxidant, detoxifies hydrogen peroxide. Decreased catalase activity has been linked with both skin carcinogenesis and progression [10], [11]. Glutathione peroxidase (GPx) has been argued to be even more crucial to maintaining cutaneous homeostasis as evidenced by a study demonstrating that a small increase in GPx activity can completely compensate for catalase-deficient fibroblasts from patients [14]. However, GPx activity has not been shown to be significantly affected by UV exposure [15], [16], [17], [18]. A decrease in vitamin C levels following UVB exposure [19] results in increased DNA damage and apoptosis [20]. Both human and animal studies have demonstrated decreased SCC formation with diets containing supplemental vitamin C [21], [22]. Previous studies of the effects of exogenous vitamin E treatment on skin carcinogenesis have resulted in a variety of observations, including a 50% decrease in skin cancer incidence with topical application of vitamin E [23] and an increase in photocarcinogenesis following treatment with more stable vitamin E esters [24]. Other studies have reported no significant association between vitamin E treatment and SCC development [25], [26], [27], [28], [29]. As vitamin E quenches free radicals, it becomes oxidized. Vitamin C is able to reduce oxidized vitamin E thus regenerating its activity; therefore, mixing vitamins E and C stabilizes topical formulations of vitamin E [30]. Ferulic acid exerts its antioxidant effects by supplying protons or hydrogen ions to free radicals with phenolic hydroxyl groups [31]. Ferulic acid also protects against the toxicity of active oxygen, or superoxide, similarly to superoxide dismutase. Ferulic acid and several of its derivatives have been shown to decrease tumor formation in chemically-induced skin carcinogenesis models [32], [33], [34], [35]. Additionally, ferulic acid further stabilizes vitamins C and E. Previously, the combination of vitamin C, vitamin E, and ferulic acid was demonstrated to have photoprotective effects when applied for four days prior to one UVB exposure [36]. Topical application of this antioxidant combination for four days prior to UVB exposure also significantly reduced UVB-induced thymine dimer formation in the epidermis 24 hours post-irradiation [37]. C E Ferulic is currently being marketed as an anti-aging treatment and sunscreen additive. However, the potential of C E Ferulic for preventing skin cancer in chronically UVB-damaged skin has not been examined.

Many cosmeceuticals are targeted primarily towards women who often have a history of considerable prior UVB exposure make use of antioxidant strategies. However, any beneficial effect of topical antioxidant application to previously sun damaged skin on skin tumor development has remained controversial. We examined the efficacy of two topical antioxidant formulations in preventing UVB-induced cutaneous SCC. Our model mimicked women who were exposed to UVB regularly in childhood and early adulthood and then markedly reduced their sun exposure and began applying topical antioxidants prior to the formation of any lesions. The current study demonstrated that topical C E Ferulic treatment effectively reduced tumor number and burden in female Skh-1 mice. Topical vitamin E treatment alone provided no preventative benefits, and in fact, resulted in accelerated tumor growth rate compared to vehicle-treated mice. This difference may be explained by the resultant increase in catalase activity levels and DNA damage present in the mice treated with vitamin E compared to those treated with C E Ferulic. Our study demonstrates both the potential detrimental effects of treating chronically UVB-damaged skin with topical vitamin E alone and the potential benefits of topically treating with a stable combination antioxidant compound for the prevention of UVB-induced SCC.

## Materials and Methods

### Ethics Statement

Outbred, female Skh-1 mice (6–8 weeks old, Charles River Laboratories, Wilmington, MA) were housed in the vivarium at The Ohio State University according to the requirements established by the American Association for Accreditation of Laboratory Animal Care. All procedures were approved by the Ohio State University Institutional Animal Care and Use Committee before the initiation of any studies (Protocol Number: 2010A00000083) and all efforts were made to minimize suffering.

### Mice

The outbred nature of this strain of mice represents the variability observed in the human population. Mice (n = 20 treated with vehicle, n = 10 treated with vitamin E, n = 10 treated with C E Ferulic) were dorsally exposed to 2240 J/m^2^ UVB, previously determined to be 1 minimal erythemal dose (MED), 3× weekly on non-consecutive days for 10 weeks. UVB dose was calculated using UVX radiometer and UVB sensor (UVP, Upland, CA) and emitted by Phillips FS40 UV bulbs (American Ultraviolet Company, Lebanon, IN). After 10 weeks of UVB exposure, the mice were treated topically with vehicle (Surgilube®; Savage Laboratories, Melville, NY), 5 mg vitamin E (d-alpha tocopherol; Sigma-Aldrich, St. Louis, MO) in vehicle, or 0.1 mL C E Ferulic (SkinCeuticals) for 15 weeks with no additional UVB exposure. This dose of vitamin E was chosen based on previously published results demonstrating efficacy in preventing UVB-induced damage [38], [39] as well as previous preliminary studies in our laboratory. Tumors larger than 1 mm in diameter were measured weekly with calipers. After sacrifice, 0.5 cm^2^ section of dorsal skin and all tumors were fixed as previously described [40] while remaining dorsal skin was snap frozen in liquid nitrogen.

### Tumor Grading

Hematoxylin and Eosin (H&E) stained tissue sections of tumors isolated from mice were graded in a blinded manner by a board-certified veterinary pathologist (DFK) as previously described [41].

### Catalase Activity Assay

Frozen dorsal skin was crushed and 15 mg was used for analysis of catalase activity using the Catalase Assay Kit (Cayman Chemical, Ann Arbor, MI) according to manufacturer’s instructions.

### Glutathione Peroxidase Activity Assay

Frozen dorsal skin was crushed and 20 mg was used for analysis of glutathione peroxidase activity using the Glutathione Peroxidase Activity Kit (Cayman Chemical) according to manufacturer’s instructions.

### Immunohistochemistry

Paraformaldehyde-fixed/OCT-embedded dorsal skin sections were cut (10 µm) onto Superfrost Plus® microscope slides (Fisher Scientific) and stored at −80°C for future analysis. Slides were thawed overnight at room temperature, baked at 60°C for 30 min, and then rehydrated in Clear Rite 3 and a graded series of ethanol. Detailed protocols for p53 and Ki67 have been described previously [42].

Slides were incubated with primary p53 antibody (clone CM5p, Novocastra (Leica Microsystems Inc.), Buffalo Grove, IL) at a 1∶500 dilution in 1× Casein at room temperature for 1 hour. p53 foci were counted as 3 or more adjacent p53-positive cells and examined in 5 fields of view at 200× magnification.

Slides were incubated with primary Ki67 antibody (Dako, Carpinteria, CA) at a 1∶200 dilution in 1× Casein overnight at 4°C in a humid chamber. Ki67-positive cells were examined in 5 fields of view at 600× magnification.

CD31: Endogenous peroxidase activity was blocked with 3% H_2_O_2_ in water for 10 minutes at room temperature. Slides were incubated in Antigen Unmasking Solution (Vector Laboratories) for 15 minutes in a microwave. After slides were cooled, they were blocked with avidin D and biotin (Vector Laboratories), each for 15 minutes, 1× Casein for 30 minutes, and incubated with primary CD31 antibody (Abcam) at a 1∶50 dilution in 1× Casein for 1 hour at room temperature. Slides were then incubated with biotinylated IgG (Vector Laboratories) at a 1∶200 dilution in 1× Casein, followed by ABC Elite. Slides were incubated in DAB solution (Vector Laboratories) for 10 minutes at RT. Slides were washed in deionized water, counterstained, and dehydrated. CD31-positive vessels were examined in 7 fields of view at 600× magnification.

### Statistical Analysis

The results presented in this paper were part of a larger experiment involving four treatment groups (of which vitamin E and C E Ferulic were two) and a single control group. Dunnett’s adjustment [43], [44] for multiplicity was used for comparing the primary outcome of tumor burden at 24 weeks between the treatment groups and control in order to restrict the probability of a type I error to 5%. The number of control mice was inflated compared to the treatment groups to increase the power of the comparison [43]. Residual plots verified the model assumptions of normality and homoscedasticity and a logarithmic transformation was utilized if necessary. Continuous outcome data were analyzed using an ANOVA approach with linear contrasts for testing the comparisons of interest. A mixed-effects regression model with a random slope and intercept by subject was used to model tumor growth from the time of tumor origination. For count data, Poisson regression was used. All analyses were conducted in SAS version 9.2 (SAS Institute, Cary, NC). *p*-values ≤0.05 were considered statistically significant.

## Results

### C E Ferulic Topical Treatment Decreased Tumor Number and Burden

To examine the effects of topical vitamin E or C E Ferulic treatment as preventative agents against tumor development, we exposed female Skh-1 hairless mice to 2240 J/m^2^ UVB (previously determined to be 1 MED in our laboratory) three times weekly for ten weeks to model chronic sun exposure. Mice were then treated topically with vehicle, vitamin E, or C E Ferulic for 15 weeks without further UVB exposure to model a lifestyle change. Non-irradiated female mice treated with either vehicle, vitamin E, or C E Ferulic did not develop tumors. Additionally, mice that were exposed to UVB but received no vehicle treatment did not exhibit a significantly different tumor burden compared to mice treated with vehicle, indicating that the vehicle had no significant effect on tumorigenesis in this study (data not shown).

**Figure 1:**
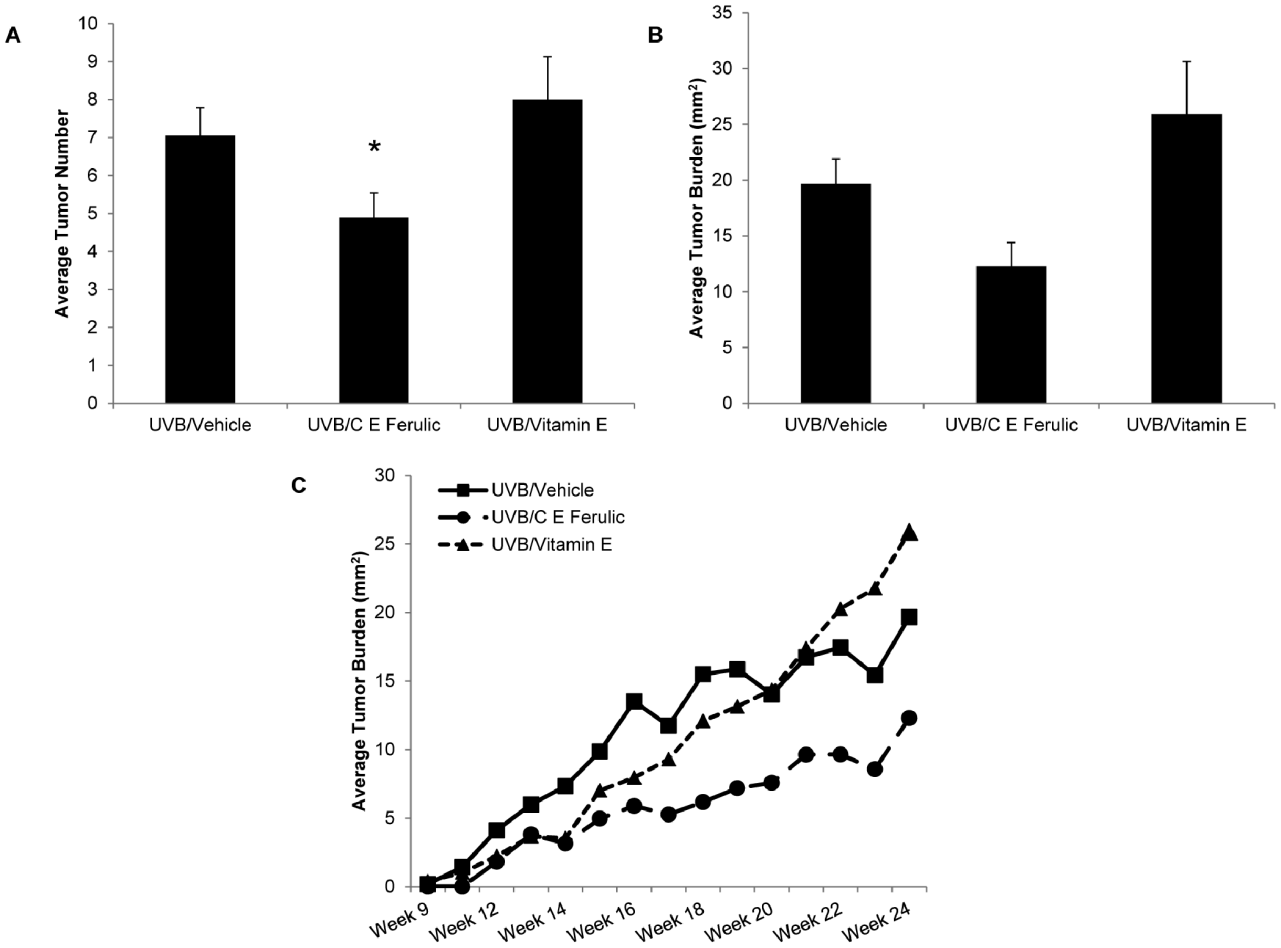
C E Ferulic decreases tumor number in female mice. Tumors with a diameter larger than 1 mm were measured weekly with calipers. Mice were exposed to 2240 J/m^2^ UVB three times weekly for 10 weeks followed by 15 weeks of topical C E Ferulic or vitamin E treatment. (A) Mice treated with C E Ferulic developed fewer tumors compared to mice treated with vehicle or vitamin E (*p = 0.0283). (B) At the end of the study, mice treated with C E Ferulic exhibited a 34% decrease in tumor burden that was not significant due to the outbred nature of this strain of mice. (C) Mice treated with vitamin E exhibited an increased growth rate compared to mice treated with vehicle or C E Ferulic that approached significance (*p* = 0.0649).

Following 10 weeks of UV exposure alone and 15 weeks of preventative topical treatment with C E Ferulic without further UVB exposures, female mice developed 30.7% fewer tumors compared to the mice treated with vehicle (*p* = 0.0340, Figure 1A). At the end of the study, female mice treated topically with C E Ferulic exhibited a 34% decrease in tumor burden compared to mice treated with vehicle (Figure 1B); however, probably as a result of variability due to the outbred nature of this strain of mice, the difference was not statistically significant (*p* = 0.6047). Mice treated topically with vitamin E demonstrated a trend toward increased tumor multiplicity with 14.9% more tumors compared to mice treated with vehicle (*p* = 0.3193, Figure 1A). Mice treated topically with vitamin E displayed a 20.7% increase in average tumor burden; the tumor burden in vitamin E-treated mice was not statistically different from that in mice treated with vehicle (*p* = 0.9566, Figure 1B).

Examining the change in tumor burden over time, we found that tumor growth rates did not significantly differ between mice treated topically with C E Ferulic and those treated with vehicle (Figure 1C). Mice treated topically with vitamin E, however, exhibited an increase in tumor growth rate compared to mice treated with vehicle, 16.6% increase in tumor burden per week versus 11.1%, respectively, which approached significance (*p* = 0.0649, Figure 1C).

### UVB-irradiated Mice Treated Topically with C E Ferulic Developed no Malignant Tumors

Tumors were isolated from mice after 10 weeks of UVB exposure followed by 15 weeks of topical treatment and scored by a board-certified veterinary pathologist (DFK). Tumors classified as papilloma were considered benign and those classified as microinvasive or fully invasive SCC were considered malignant. As seen in Table 1, female mice treated with vehicle developed papillomas, microinvasive SCC, and fully invasive SCC. Female mice preventatively treated with C E Ferulic developed only papillomas. Mice preventatively treated with vitamin E developed both papillomas and microinvasive SCC, with a lower percentage of malignant tumors compared to vehicle-treated mice.

**Table 1:** Distribution of benign and malignant tumors.

	% Benign Tumors	% Malignant Tumors
UVB/Vehicle	89.7	10.3
UVB/C E Ferulic	100.0	0.0
UVB/Vitamin E	97.1	2.9

### Topical Vitamin E Treatment Increased the Number of p53-positive Foci

**Figure 2:**
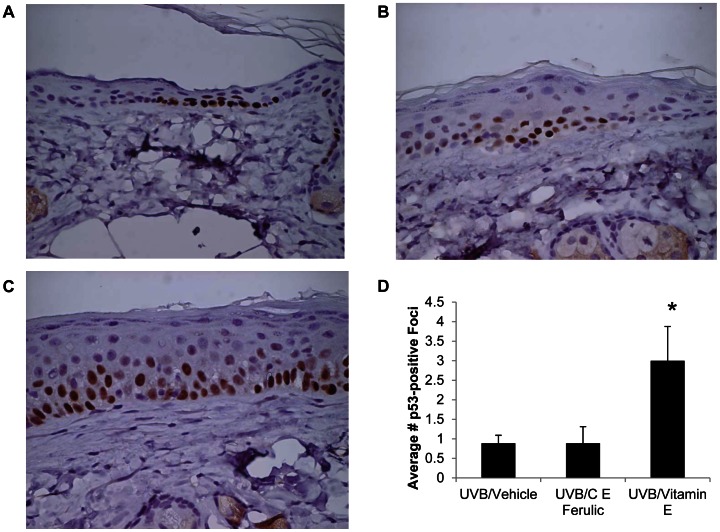
Mice treated with topical vitamin E have increased numbers of p53-positive foci. Dorsal, tumor-free skin sections were examined via immunohistochemistry with an antibody detecting both wild type and mutant p53. Representative images of skin from mice treated with (A) vehicle, (B) C E Ferulic, and (C) vitamin E for 15 weeks after 10 weeks of UVB exposure. (D) The average number of p53-positive foci per field of view was significantly higher in mice treated with vitamin E compared to vehicle-treated mice (*p = 0.0216).

As a measure of total DNA damage, tumor-free, dorsal skin sections were examined for p53-positive foci via immunohistochemistry. Because the antibody used detected both wild type and mutant p53, some p53-positive foci represented expanding clones of keratinocytes with mutated p53. The density of p53-positive foci in hairless mice has been shown to correlate well with skin tumor risk [45]. The mean number of p53 foci was not significantly altered with topical C E Ferulic treatment (Figure 2A) compared to vehicle-treated mice (Figure 2B). The average number of p53-positive foci was significantly increased with preventative topical vitamin E treatment in female skin (*p* = 0.0216, Figure 2C, quantified in Figure 2D), suggesting a greater risk of skin tumors in mice treated with vitamin E alone.

### Topical Vitamin E Treatment Increased the Number of CD31-positive Blood Vessels

**Figure 3:**
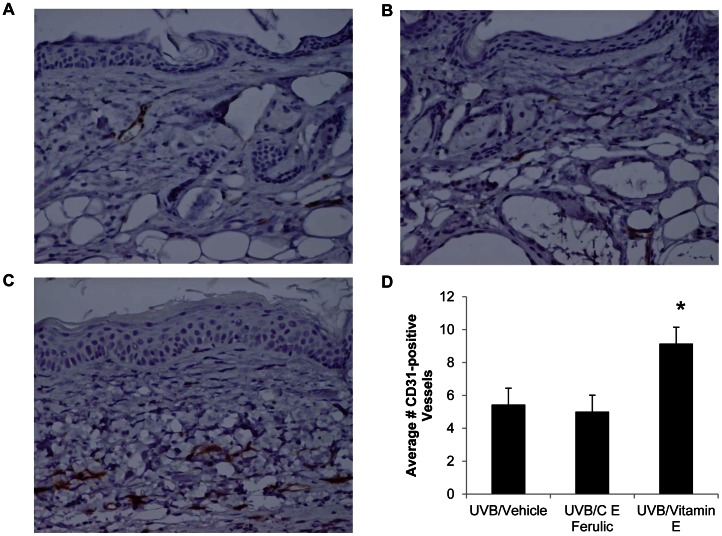
Mice treated with topical vitamin E have increased numbers of CD31-positive vessels. Dorsal, tumor-free skin sections were examined for CD31-positive vessels via immunohistochemistry. Representative images of skin from mice treated with (A) vehicle, (B) C E Ferulic, and (C) vitamin E for 15 weeks after 10 weeks of UVB exposure. (D) The average number of CD31-positive vessels per field of view was significantly higher in mice treated with vitamin E compared to vehicle-treated mice (*p<0.0001).

To examine changes in vasculature, tumor-free dorsal skin sections were examined for CD31-positive blood vessels via immunohistochemistry. Several studies have demonstrated a link between microvessel density, tumor growth, and metastasis [46], [47], [48]. The mean number of CD31-positive blood vessels was not significantly altered with topical C E Ferulic treatment (Figure 3A) compared to vehicle-treated mice (Figure 3B). However, the average number of blood vessels was significantly increased with topical vitamin E treatment (*p*<0.0001, Figure 3C, quantified in Figure 3D).

### Vitamin E Topical Treatment Increased Cutaneous Proliferation

**Figure 4:**
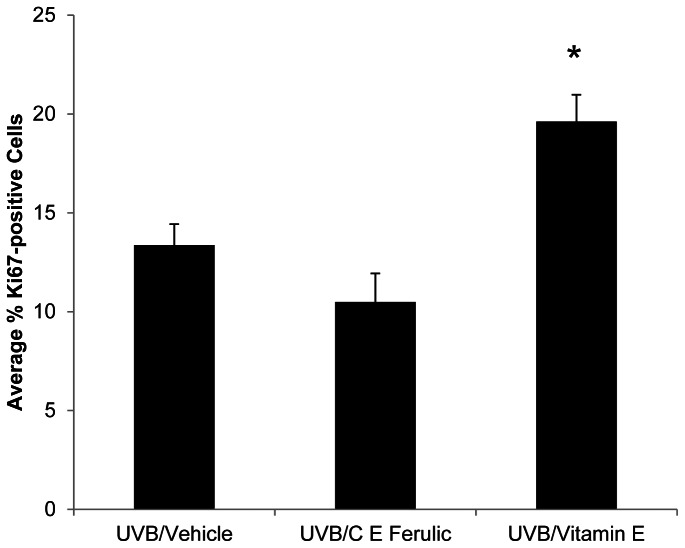
Female skin treated with vitamin E displays increased levels of proliferation. Dorsal, tumor-free skin sections were stained for Ki67. Mice treated with vitamin E exhibited higher percentages of Ki67-positive cells compared to mice treated with vehicle or mice treated with C E Ferulic (*p = 0.0004).

To examine changes in proliferation rates, tumor-free, dorsal skin sections were stained for Ki67. The percentage of Ki67-positive cells was not significantly altered in C E Ferulic-treated skin compared to vehicle-treated skin after 10 weeks of UVB exposure followed by 15 weeks of topical treatment with no additional UVB exposure. In contrast, the percentage of Ki67-positive cells exhibited a significant increase with topical vitamin E treatment compared to vehicle-treated mice (*p* = 0.0004, Figure 4).

### Topical Vitamin E Treatment Increased Catalase and GPx Activity Levels

**Figure 5:**
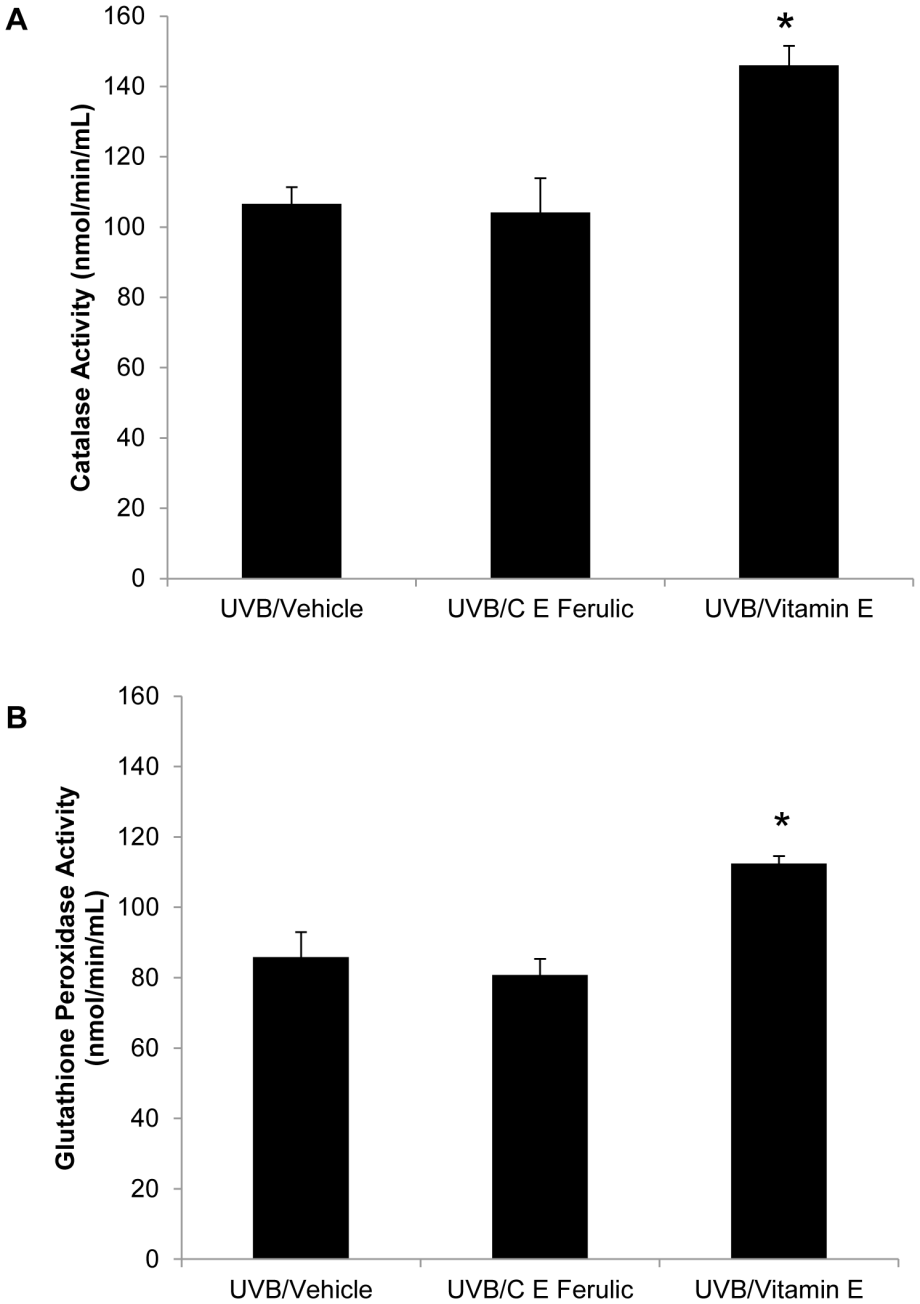
Vitamin E increases cutaneous catalase and glutathione peroxidase activity levels. Protein extracted from frozen, crushed skin was examined for antioxidant activity. (A) Compared to mice treated with vehicle or C E Ferulic, topical vitamin E significantly increased catalase activity levels in female skin (*p<0.0001). (B) Mice treated with vitamin E exhibited elevated glutathione peroxidase activity (*p = 0.0125).

Protein was extracted from tumor-free, dorsal skin in order to examine catalase activity levels. Catalase activity levels in skin treated topically with C E Ferulic for 15 weeks after stopping UVB exposures were not significantly altered compared to those observed in vehicle-treated skin (*p* = 0.7631, Figure 5A). In contrast, catalase activity levels were significantly increased in female skin treated with vitamin E compared to vehicle-treated skin (*p*<0.0001, Figure 5A). Likewise, GPx activity levels were increased with topical vitamin E treatment (*p* = 0.0125, Figure 5B) while mice treated topically with C E Ferulic did not exhibit a significant alteration in GPx activity (*p* = 0.6415, Figure 5B). Recently, decreased catalase activity was linked with increased tumor burden, while increased catalase activity corresponded with a decrease in tumor burden in male Skh-1 mice [40]. This link was not observed in female mice [42].

## Discussion

Because the skin is constantly exposed to external stimuli, it has developed a system of endogenous antioxidants to deal with environmental challenges. Antioxidants have been shown to effectively inhibit the initiation phase of carcinogenesis by detoxifying carcinogens, especially in chemically induced models [49]. Antioxidants also have an inhibitory role in the promotion phase where they scavenge ROS and work to prevent antioxidant network depletion [49].

Previously, we demonstrated that treating female Skh-1 mice with a topical anti-inflammatory drug decreased DNA damage, tumor number and tumor grade [50]. In the current study we showed that topically applied C E Ferulic, a stable antioxidant compound, protects chronically UVB-damaged skin against skin tumor development. Tumor number and burden were decreased in C E Ferulic-treated mice compared to vehicle-treated mice. Confirming the results of several previous studies [25], [26], [27], [28], [29], topical vitamin E provided no beneficial effects with regard to tumor burden; in fact, topical vitamin E alone resulted in increased tumor number and burden as well as increased DNA damage as indicated by p53 stabilization. Additionally, topical vitamin E treatment contributed to increased proliferation of epidermal cells, as well as an increase in angiogenesis. Interestingly, catalase and glutathione peroxidase activity were increased only with vitamin E treatment.

Topical vitamin E increased the number of p53-positive foci observed in the epidermis, reflecting an increase in DNA damage. If vitamin E is exerting a pro-oxidant effect, increased alpha tocopheroxyl radicals may be contributing to increased levels of DNA damage. However, previous studies indicate that both vitamins E and C decrease the amount of UVB-induced oxidative DNA damage, specifically 8-hydroxy-2-deoxyguanosine adducts [20], [51]. Because we saw no change in the level of overall DNA damage in C E Ferulic-treated mice but elevated levels of DNA damage in vitamin E-treated mice, it is possible that the concentrations of the vitamins utilized, as well as the delivery and treatment schedule, may play important roles in determining the efficacy of these antioxidants.

ROS, including alpha tocopheroxyl radicals, contribute to oxidative stress after UVB exposure. Previous studies suggested that alpha tocopheroxyl radicals generated from alpha tocopherol play a pivotal role in antioxidant-induced angiogenesis [52]. In the current study, the increased vessel density observed in vitamin E-treated mice further supports the mounting evidence that oxidative stress can act as a trigger for angiogenesis. Interestingly, increased levels of vitamin C have been demonstrated to prevent the increased angiogenesis observed with high vitamin E treatment concentrations; the suggested mechanism is scavenging of alpha tocopheroxyl radicals [53]. While we only observed a change in angiogenesis with the vitamin E treatment, it is possible that the C E Ferulic is scavenging tocopheroxyl radicals at a rate that prevents an increase in angiogenesis but is not sufficient to cause a decrease.

It is important to note that mice treated with either C E Ferulic or vitamin E developed lower percentages of malignant tumors compared to vehicle-treated mice. While mice treated topically with C E Ferulic exhibited decreased tumor multiplicity and burden, mice treated topically with vitamin E alone developed an increased tumor number and burden compared to vehicle-treated mice. It may be possible that vitamin E alone is more effective in the late phase of tumorigenesis thus affecting tumor progression but not tumor development as compared to C E Ferulic. This may explain why, while we did not observe a decrease in tumor number or burden in mice treated with vitamin E, we did see a smaller percentage of malignant tumors in mice treated with vitamin E compared to vehicle-treated mice. It is important to note, however, that due to the outbred nature of this strain of mice these differences were not statistically significant.

Of the antioxidants, catalase has been the best studied in terms of its role in cutaneous homeostasis. Recently, we demonstrated an important link between restoring catalase activity after chronic UVB exposures and decreasing tumor burden in male but not female mice [40], [42]. In the current study, the observed increase in catalase activity in female mice treated with vitamin E suggests that this antioxidant may actually be acting as a pro-oxidant. Antioxidant activity and efficacy depend heavily on the preexisting redox status of the environment [53], with several studies demonstrating that vitamin E may exert a pro-oxidant effect when it is administered under low or even normal levels of oxidative stress both *in vitro* [54], [55] and in vivo [56], [57]. Although we did not specifically measure oxidative stress in the current study, it is possible that low levels of oxidative stress in the skin following the cessation of UVB exposure resulted in the pro-oxidant effect with the application of the vitamin E treatment. The fact that mice treated with C E Ferulic did not exhibit increased catalase activity levels but did demonstrate decreased tumor number and burden, while mice treated with vitamin E exhibited increased catalase activity levels and no beneficial effects on tumor burden, suggests that the observed increased catalase activity levels were not beneficial for decreasing tumor burden in female mice.

GPx most often functions as an effective antioxidant and tumor preventative agent, as evidenced by several studies demonstrating that impaired GPx activity correlates with the development of various human cancers [58], [59], [60]. However, higher levels of GPx have also been found to have procarcinogenic activity as demonstrated by enhanced skin tumor incidence in transgenic mice overexpressing Gpx1 [61]. This finding was further supported by studies demonstrating that GPX1 [62] and GPX2 [63] are able to inhibit stress-induced apoptosis. In the current study, mice treated topically with vitamin E alone exhibit enhanced GPx activity, which correlates with enhanced overall DNA damage and cutaneous proliferation, and ultimately the increased formation of skin tumors. However, because the cutaneous antioxidant networks are exceedingly complex, further studies are needed to fully understand the interactions among the different endogenous antioxidants in the skin, as well as with exogenously applied antioxidants.

With the ever-increasing skin cancer incidence, antioxidant supplementation in food, sunscreens, and lotions has become widespread. While some animal models suggest beneficial effects from antioxidants, it is important to note that antioxidants are delivered prior to any UV exposure in many of these studies [19], [23]. Clinical trials examining potential effects of antioxidant supplementation have yielded contradictory results. The Supplementation in Vitamins and Mineral Antioxidants (SU.VI.MAX) study revealed that daily supplementation with nutritional doses of antioxidants decreased the overall incidence of cancer in men, but had no effect in women [64]. Further, the impact of antioxidant supplementation on skin cancer incidence, specifically, was examined within the framework of the SU.VI.MAX study [65], revealing an increased incidence of skin cancer in women and a trend towards decreased skin cancer in men receiving antioxidant supplements. The current study supports these findings in that, as reported in the SU.VI.MAX study, there is a trend towards increased tumor burden in female mice treated with vitamin E, a single antioxidant. In contrast, the group treated with C E Ferulic, a combination antioxidant exhibited decreased tumor number.

The delivery method of antioxidant supplements also seems to play a role in the study outcomes. A systematic review of randomized controlled trials reported that there was no beneficial effect of oral vitamin or antioxidant supplementation on skin cancer prevention but that topical antioxidant application did offer some degree of protection in high risk individuals [66]. Our current study supported these findings, in that mice with chronically UVB-damaged skin that were treated topically with C E Ferulic had decreased numbers of skin tumors compared to vehicle-treated mice. Further, the antioxidant concentration and activity in the various products varied greatly; thus, standardized testing and labeling will be required to allow consumers to more easily compare these products [67]. Further studies are needed to understand antioxidant activity *in vivo* and to measure topical antioxidant efficacy.

In summary, we have shown in a model of UVB-induced SCC that topically treating female Skh-1 hairless mice with C E Ferulic for 15 weeks after 10 weeks of UVB exposure decreased tumor number and burden and suppressed the formation of malignant tumors. In contrast, treating with topical vitamin E had no therapeutic benefits, and, in fact, resulted in increased overall DNA damage and vessel density, and tended to increase tumor number, burden and growth rate, possibly due to the pro-oxidant effects of vitamin E supplementation to chronically UVB-damaged skin.

Because of the focus on antioxidant supplementation in many products targeted towards women who have often been exposed to significant amounts of UVB, these findings are especially relevant. Overall, our data suggest that topically treating chronically UVB-damaged skin with 5% vitamin E alone may actually promote SCC development. Our findings may help explain previous contradictory evidence regarding antioxidant supplementation and cancer incidence.
